# Plate fixation of the anterior pelvic ring in patients with fragility fractures of the pelvis

**DOI:** 10.1007/s00068-021-01625-z

**Published:** 2021-03-11

**Authors:** Michiel Herteleer, Mehdi Boudissa, Alexander Hofmann, Daniel Wagner, Pol Maria Rommens

**Affiliations:** 1 Department of Orthopaedics and Traumatology, University Medical Center Mainz, Mainz, Germany; 2 Department of Traumatology and Orthopaedics, Westpfalz-Clinics Kaiserslautern, Kaiserslautern, Germany

**Keywords:** Fragility fracture pelvis, Double plate osteosynthesis, Single plate osteosynthesis, Geriatric trauma

## Abstract

**Introduction:**

In fragility fractures of the pelvis (FFP), fractures of the posterior pelvic ring are nearly always combined with fractures of the anterior pelvic ring. When a surgical stabilization of the posterior pelvis is performed, a stabilization of the anterior pelvis is recommended as well. In this study, we aim at finding out whether conventional plate osteosynthesis is a valid option in patients with osteoporotic bone.

**Materials and methods:**

We retrospectively reviewed medical charts and radiographs of all patients with a FFP, who underwent a plate osteosynthesis of the anterior pelvic ring between 2009 and 2019. Patient demographics, fracture characteristics, properties of the osteosynthesis, complications and revision surgeries were documented. Single plate osteosynthesis (SPO) at the pelvic brim was compared with double plate osteosynthesis (DPO) with one plate at the pelvic brim and one plate anteriorly. We hypothesized that the number and severity of screw loosening (SL) or plate breakage in DPO are lower than in SPO.

**Results:**

48 patients with a mean age of 76.8 years were reviewed. In 37 cases, SPO was performed, in 11 cases DPO. Eight out of 11 DPO were performed in patients with FFP type III or FFP type IV. We performed significantly more DPO when the instability was located at the level of the pubic symphysis (*p* = 0.025). More patients with a chronic FFP (surgery more than one month after diagnosis) were treated with DPO (*p* = 0.07). Infra-acetabular screws were more often inserted in DPO (*p* = 0.056). Screw loosening (SL) was seen in the superior plate in 45% of patients. There was no SL in the anterior plate. There was SL in 19 of 37 patients with SPO and in 3 of 11 patients with DPO (*p* = 0.16). SL was localized near to the pubic symphysis in 19 of 22 patients after SPO and in all three patients after DPO. There was no SL in DPO within the first month postoperatively. We performed revision osteosynthesis in six patients (6/48), all belonged to the SPO group (6/37). The presence of a bone defect, unilateral or bilateral anterior pelvic ring fracture, post-operative weight-bearing restrictions, osteosynthesis of the posterior pelvic ring, and the presence of infra- or supra-acetabular screws did not significantly influence screw loosening in SPO or DPO.

**Conclusion:**

There is a high rate of SL in plate fixation of the anterior pelvic ring in FFP. In the vast majority, SL is located near to the pubic symphysis. DPO is associated with a lower rate of SL, less severe SL and a later onset of SL. Revision surgery is less likely in DPO. In FFP, we recommend DPO instead of SPO for fixation of fractures of the anterior pelvic ring, which are located in or near to the pubic symphysis.

## Introduction

Fragility fractures of the pelvis (FFP) occur as a result of low-energy trauma and exhibit different morphological characteristics. The majority of FFP have non- or minimally displaced fracture components in the anterior and posterior part of the pelvic ring. The degree of instability may increase over time due to fracture progression [1]. Surgical fixation is performed in displaced fractures to diminish pain, to allow early active rehabilitation and to obtain uneventful fracture healing [2]. Operative treatment is primarily focused on stabilization of the posterior pelvic ring.

In FFP, the anterior pelvic ring is fractured in up to 97% [3, 4]. These anterior fractures are painful and contribute to the overall loss of stability of the pelvic ring. Surgical stabilization of any anterior pelvic fracture should be considered whenever a fixation of the posterior pelvis is performed [5]. Experience with operative fixation of the anterior pelvic ring is available from pelvic disruptions after high-energy trauma. Numerous fixation methods have been described: external fixation, internal fixation, plate osteosynthesis and retrograde transpubic screw fixation. Plate osteosynthesis is standard of care in case of pubic diastasis or in fractures that are localized near to the symphysis [6]. Plate fixation creates high stability and reduces motion in the joint or at the fracture site. The plate bridges the pubic symphysis and is subject to normal physiological motion, which may result in plate breakage, screw breakage or screw loosening [7–9]. Several authors report implant failures from 11% up to 90%, but the need for surgical revision due to major complaints is low [10–13].

Surgical fixation of the anterior pelvic ring in FFP can be performed with the same methods as in high-energy pelvic disruption. Up to one third of fractures of the anterior pelvic ring in FFP are located in the pubic bone close to the symphysis [2]. Plate osteosynthesis seems to be the most valid option in these cases. Nevertheless, due to low bone mineral density, there is a diminished holding power of the screws, which is an additional risk factor for implant failure.

The primary aim of this study is to evaluate plate osteosynthesis of the anterior pelvic ring in patients with FFP by assessing the amount of implant-related problems and their risk factors. The secondary aim is comparing single plate osteosynthesis (SPO) with double plate osteosynthesis (DPO).

## Patients and methods

### Population

We retrospectively reviewed the medical charts and radiological data of all patients with FFP, who underwent a plate osteosynthesis of the anterior pelvic ring between 2009 and 2019 (11-year period). Patients with plate osteosynthesis of the anterior pelvis in pelvic fractures due to a high-energy trauma were excluded.

### Fracture characteristics

The fractures were classified in accordance to the FFP classification of Rommens and Hofmann, which mainly focuses on the instability of the posterior pelvic ring [4]. The pattern of the instability of the anterior pelvic ring was described as unilateral, bilateral or located at the pubic symphysis. The localization of the fractures was classified according to the Nakatani fracture classification, which distinguishes between fractures of the pubic bone (Nakatani I), fractures of the superior pubic ramus above the obturator foramen (Nakatani II) and fractures lateral to the obturator foramen (Nakatani III) [14]. Fracture displacement was described as non-displaced, minor (< width of the pubic ramus) or major (> width of the pubic ramus). FFP was classified as acute or chronic (operative treatment more or less than one month after diagnosis). Each fracture was also assessed for the presence of a bone defect.

### Surgical technique

In all patients with a fracture of the posterior pelvic ring (FFP II, FFP III and FFP IV), the posterior fixation was performed in the first phase of the surgical procedure. Surgical fixation of an iliac wing fracture was done through the first window of the ilioinguinal approach. Fractures of the sacrum and sacroiliac joint were stabilized with minimal invasive techniques. Any minimal invasive fixation of the posterior pelvis was carried out in prone position and the patient turned to the supine position consecutively. All fixations of the anterior pelvis were performed through the modified Stoppa approach.

### Properties of SPO and DPO osteosynthesis

Whether a SPO or DPO was carried out depended on the surgeon’s choice. The unique or superior plate was always a pelvic brim plate. Non-angular as well as angular stable small fragment pelvic reconstruction plates (DePuy Synthes, Umkirch, Germany) were used. A minimum of two screws on each side of the fracture  were inserted. Screws were as long as possible within their bony corridors. On the postoperative radiographs, the number and location of the inserted screws were registered. We distinguished between screws located in the pubic bone (Nakatani I), in the superior pubic ramus (Nakatani II), in the infra-acetabular corridor—which is the bone corridor running from the iliopectineal eminence to the ischial tuberosity**—**(Nakatani III) and screws inserted above the acetabulum. The cumulative length of all screws was calculated.

**Fig. 1:**
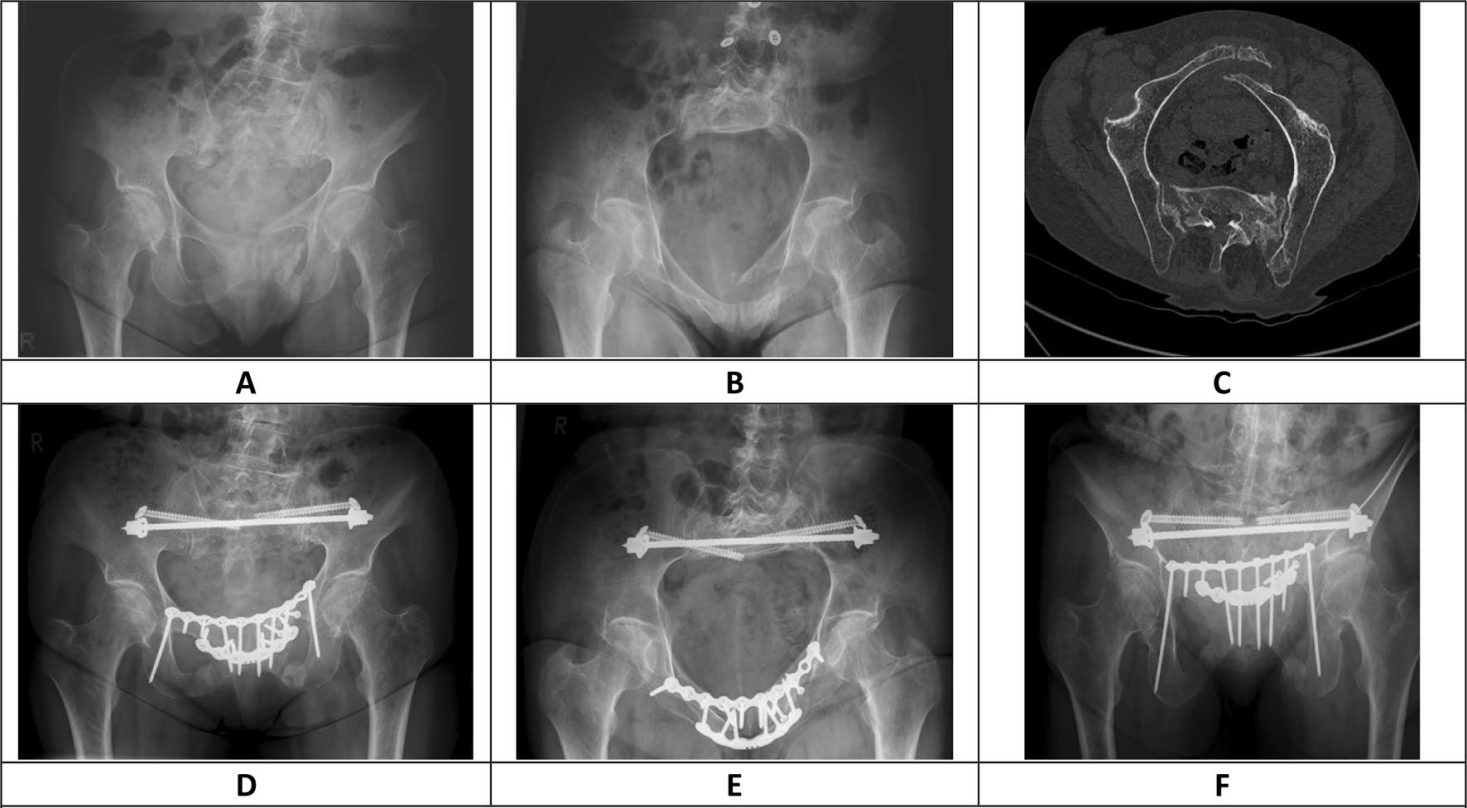
Shows an 83 year old female patient with a FFP 4b and a largely displaced fracture in the Nakatani II region of the anterior pelvic ring. (**a** Conventional ap radiograph, **b** conventional inlet radiograph, **c** CT reconstruction in the pelvic brim view). The posterior pelvic ring was treated with a sacral bar and bilateral SI screws. The anterior fractures was treated with a DPO (superior ten-hole non-angular stable plate with bilateral infra-acetabular screws, two lag screws, six-hole angular stable anterior plate). This patient did not have any screw loosening within the follow-up period of 12 months. (**d** Conventional ap radiograph, **e** conventional inlet radiograph, **f** conventional outlet radiograph)

**Fig. 2:**
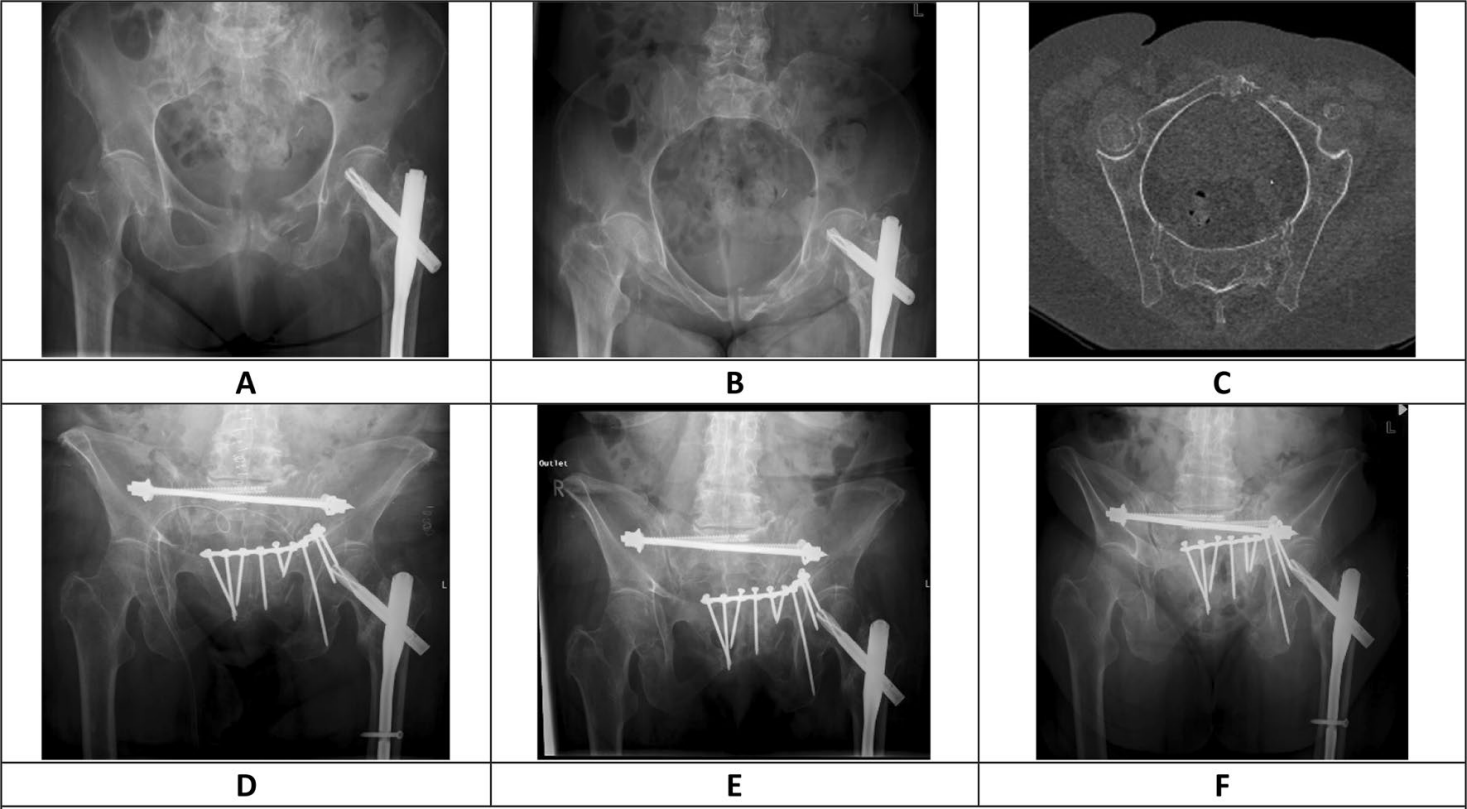
Shows a 85 year old patient with a chronic FFP 4b fracture with a largely displaced fracture of left anterior pelvic ring in the Nakatani II region. (**a** Conventional AP radiograph, **b** conventional inlet radiograph **c** CT reconstruction pelvic brim view). She was treated with a sacral bar and bilateral SI screws posteriorly and a ten-hole SPO with unilateral infra- and supra-acetabular screws. The first screw loosening was observed within the first week after treatment (**d** 1 week post-operative conventional outlet radiograph) and progressive loosening of supplementary screws over the following 4 months. (**e** 4 weeks post-operative conventional outlet radiograph, **f** 4 months post-operative conventional outlet radiograph). The fracture healed and the patient did not require any revision surgery

The second plate was always an angular stable symphysis plate (DePuy Synthes, Umkirch, Germany), which was placed anteriorly with the screw direction from anterior to posterior (Figs. 1 and 2). Depending on the surgeon`s estimation of the postoperative stability, an immediate postoperative weight-bearing or non-weight-bearing protocol was initiated.

#### Screw loosening (SL) and plate breakage

We assessed the outcome of the plate fixation on all available postoperative radiographs and CT data. Type, localization, time delay after surgery and rate of complications as well as the number of surgical revisions, which were directly related to the plate osteosynthesis, were registered. We distinguished between screw loosening (SL), screw and plate breakage. SL was scored as minimal (screw head only), moderate (more than screw head until less than half of screw length) or severe (more than half of screw length). The localization of the complication was classified in accordance to the Nakatani classification [14].

We performed different sub-analyses to evaluate if SL or plate breakage was associated with the following factors: the localization of the fracture (in accordance with the Nakatani classification), the instability being unilateral, bilateral or at the pubic symphysis, the cumulative length of screws used in the upper plate, the use of infra-acetabular or supra-acetabular screws, the presence of a bone defect at the fracture site or early postoperative weight-bearing.

Finally, we compared the patients with SPO with those with DPO and hypothesized that the number and severity of complications in DPO will be lower than in SPO.

### Statistical analysis

All data were analyzed using the Statistical Package for Social Sciences (SPSS) version 26.0 (SPSS, Chicago, Illinois, USA). Descriptive analysis was performed for all variables and differences between groups were calculated using the Fischer’s exact test for categorical data and the Mann–Whitney *U* test for continuous data. Normality of continuous data was tested using the Shapiro–Wilk test. Statistical significance was set at *p* < 0.05.

## Results

### Patient characteristics and fracture properties (demographics)

We identified 48 patients of which 46 were female and 2 were male. The mean age was 76.8 (± 8.2 SD) years for female patients and 81 (± 7.1 SD) years for male patients. Two patients had FFP Type I, 11 had FFP Type II, 9 had FFP Type III and 26 patients had FFP Type IV fractures (Table 1). As a whole, there were 46 FFP with involvement of the posterior pelvis and two without. The instability was unilateral in 28 patients, bilateral in 14 and at the pubic symphysis in 4 patients. In our 48 patients, 61 fractures of the anterior pelvis were identified of which only 13 were non-displaced, 17 showed minor and 31 major displacement. According to the Nakatani classification, 34 fractures were localized in zone 1, 18 in zone 2 and 6 in zone 3. A bone defect was present at the fracture site in 10 of our patients. Of all our patients, 15 had an acute and 33 a chronic FFP. 24 patients (19 SPO and 5 DPO) were allowed full weight-bearing immediate postoperative and 24 patients (18 single plates and 6 double plates) were only allowed to do short transfers for 6 weeks. The mean follow-up was 35 months (SD: 31 months, range 2–148 months) for the entire cohort, 31 months (SD: 23 months, range 3–81 months) for the SPO group and 49 months (SD: 47 months, range 2–148 months) for the DPO group.

**Table 1:** Description of the demographic differences and differences in fracture morphology between patients treated with a SPO and DPO

	Total	Single	Double	Significance
Patients	48	37	11	
Age (mean)		76.5	77.6	*p* = 0.687
Fracture type				
Type 1				
FFP Ia	0	0	0	*p* = 0.646
FFP Ib	2	2	0	
Type 2				
FFP IIa	0	0	0	
FFP IIb	4	4	0	
FFP IIc	7	4	3	
Type 3				
FFP IIIa	4	4	0	
FFP IIIb	0	0	0	
FFP IIIc	5	4	1	
Type 4				
FFP Iva	0	0	0	
FFP IVb	16	11	5	
FFP IVc	10	8	2	
Localisation of instability				
Unilateral	28	22	6	*p* = 0.025
Bilateral	14	13	1	
Pubic fymphysis	6	2	4	
Localisation of fractures				
Nakatani I	34	26	8	
Nakatani II	18	17	1	
Nakatani III	6	5	1	
Symphysis	3	2	1	
Fracture displacement				
Undisplaced	13	9	4	*p* = 0.7
Minor displacement	17	13	4	
Major displacement	31	26	5	
Bone defect				
No	38	28	10	*p* = 0.416
Yes	10	9	1	
Acute vs chronic				
Acute	15	14	1	*p* = 0.070
Chronic	33	23	10	
Screw type superior plate				
Infra-acetabular	39	30	9	*p* = 0.056
Unilat	16	15	1	
Bilateral	23	15	8	
Supra-acetabular	14	11	3	
Unilat	14	11	3	
Bilateral	0	0	0	
Weight bearing				
No restrictions	24	19	5	*p* = 1
Short transfers	24	18	6	

#### Fixation of the posterior pelvic ring

Fixation of the posterior pelvic ring was performed in all patients with FFP II, FFP III and FFP IV (*n* = 46/48 patients). In 34 patients, we performed a transsacral bar osteosynthesis in combination with uni- or bilateral sacro-iliac screws (Fig. 1). In five patients, a transiliac internal fixator in combination with iliosacral screws was used. In five patients, a plate or screw fixation of the iliac wing was performed through the first window of the ilioinguinal approach. In two patients, only iliosacral screws were inserted (Table 2).

**Table 2:** Comparison of the complications between single and double plate osteosynthesis

	Total	Single	Double	Significance
Screw loosening superior plate				
No	26	18	8	*p* = 0.16
Yes	22	19	3	
Time of screw loosening				
Within first week	9	3	0	*p* = 0.19
Between 1st week and 1st month	3	3	0	
After 1st month	10	7	3	
Localisation of screw loosening				
Nakatani I	10	8	2	*p* = 0.843
Nakatani I + II	7	6	1	
Nakatani I + III	2	2	0	
Nakatani II	1	1	0	
Nakatani III	1	1	0	
Nakatani I + II + III	1	1	0	
Degree of screw loosening				
No	24	18	8	*p* = 0.361
Minimal	8	6	2	
Moderate	8	7	1	
Severe	6	6	0	
No-minimal	34	24	10	*p* = 0.14
Moderate-severe	14	13	1	
Plate breakage superior plate				
Yes	4	3	1	*p* = 1
No	44	34	10	
Revision osteosynthesis				
Yes	6	6	0	*p* = 0.31
No	42	31	11	

### Single Plate Osteosynthesis (SPO) vs Double Plate Osteosynthesis (DPO)

The demographics of the patients with SPO vs. DPO are shown in Table 1. We performed 37 SPO and 11 DPO. Eight out of 11 DPO were performed in patients with FFP Type III or Type IV. The median length of the superior plate was 10 holes (range 5–16 holes) in SPO and 10 holes (range 4–12 holes) in the DPO (Fig. 1). A 4-hole plate was only used in one patient (DPO group), a six-hole plate in seven patients (6 SPO group and 1 DPO group). The average cumulative screw length in SPO was 434 mm and 481 mm in DPO (*p* = 0.38). We performed significantly more DPO when the instability was located at the level of the pubic symphysis (*p* = 0.025). There was no significant correlation between the use of SPO or DPO and the FPP classification (*p* = 0.646). There was a trend towards more patients with a chronic FFP being treated with a DPO (*p* = 0.07). There was also a trend towards more bilateral infra-acetabular screws (superior plate) being used in DPO (*p* = 0.056).

#### SL and plate breakage in SPO and DPO

**Fig. 3:**
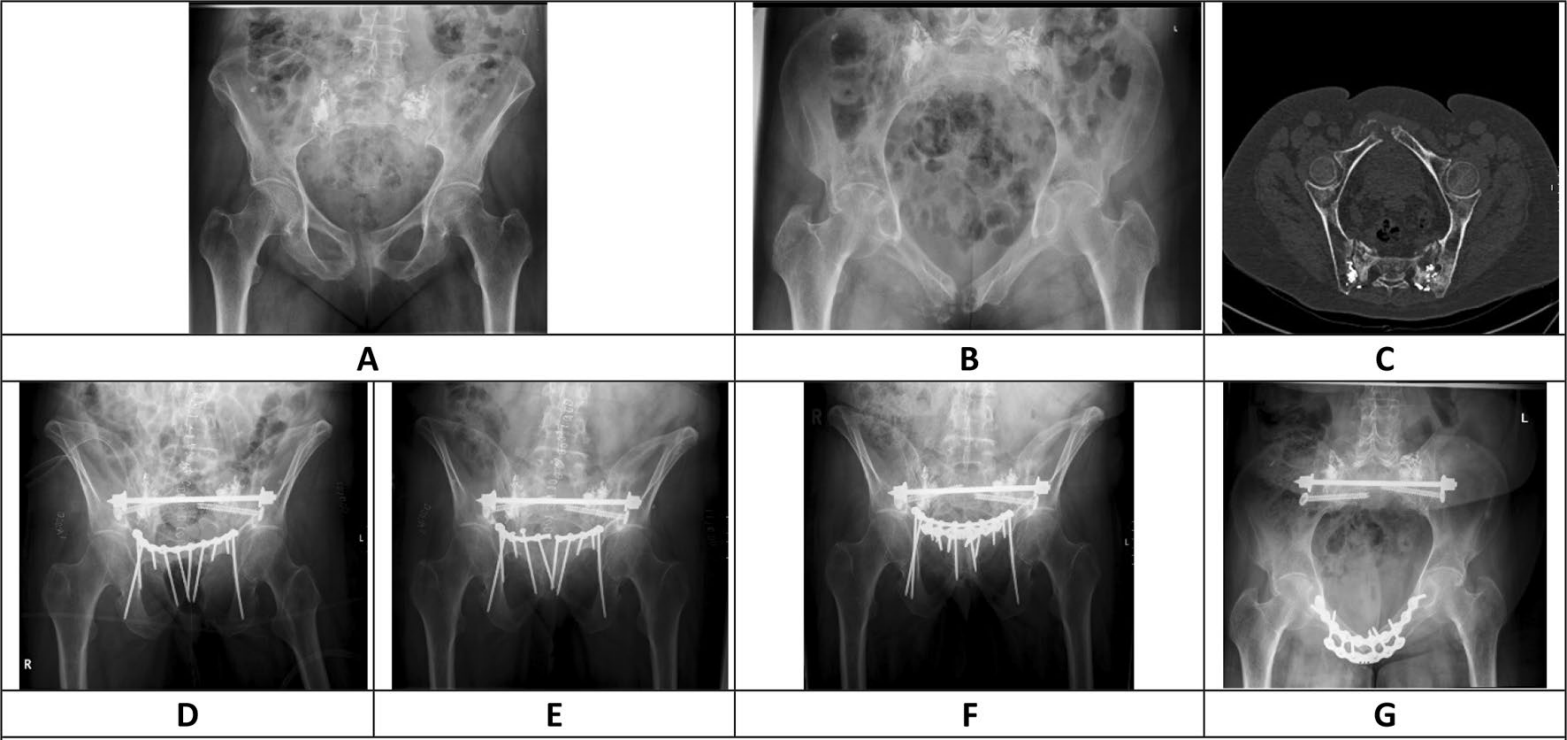
Shows a 67 year old patient who was treated with a sacroplasty elsewhere but transferred to our center due to fracture progression. She presented with a FFP 4c fracture and anterior instability of the pubic symphysis extending into the Nakatani I region 91 days after the injury. (**a** Conventional ap radiograph, **b** conventional inlet radiograph, **c** CT reconstruction in pelvic brim view). The fracture of the anterior pelvic ring was located at the level of the symphysis and extending into the right Nakatani I region. The chronic fracture was initially treated with a sacral bar and bilateral SI screws posteriorly and a ten-hole SPO with bilateral infra-acetabular screws. (**d** Immediate post-operative conventional outlet radiograph). The plate broke 9 days after the initial operation (**e** 10 day post-operative conventional outlet radiograph) and a revision DPO was performed with an additional anterior six-hole angular stable plate. A follow-up of 4 months was available and during this time period no screw loosening was observed. (**f** Conventional outlet radiograph 4 months post-operative, **g** conventional inlet radiograph 4 months postoperative)

Table 3 provides an overview of the complications, which are related to the plate osteosynthesis. SL was observed in 22/48 patients of our population. SL was only seen in the superior plate (22 patients) but not in the anterior plate (Fig. 2). There was more SL in SPO (19 out of 37 patients) than in DPO (3 out of 11 patients) but this difference was not statistically significant (*p* = 0.16). There was no significant correlation between the FFP—classification and the occurrence of SL in both the SPO group and the DPO group. We observed 12 SL in SPO within the first postoperative month, but none in DPO (Table 3). Nevertheless, this difference was not significant (*p* = 0.19). SL was observed in 19 out of 22 patients in the Nakatani I region. We observed moderate to severe screw loosening in 13 patients with SPO and in only one patient with DPO. This difference was not statistically significant (*p* = 0.14). No revision osteosynthesis was performed in DPO whereas six revisions were necessary in the single plate group (6/37) (*p* = 0.31). In three cases, a revision less than one month after surgery was performed due to early implant loosening with complete loss of stability. In the other three cases, the revisions were performed later: once due to an additional peri-implant fracture, once due to symptomatic plate breakage and once due to severe screw loosening. Plate breakage was observed in the superior plate only: three times in SPO and once in DPO (Fig. 3). The presence of a bone defect, unilateral or bilateral anterior pelvic ring fracture, post-operative weight-bearing, osteosynthesis of the posterior pelvic ring, the type of osteosynthesis of the posterior ring and the presence of infra- or supra-acetabular screws in the pelvic brim plate did not significantly influence screw loosening in SPO or DPO (Table 3).

**Table 3:** Comparison of relation between screw loosening, fracture properties and properties of the osteosynthesis

Screw loosening	Total			SPO group			DPO group		
	No SL	SL		No SL	SL		No SL	SL	
Bone defect			*p* = 0.31			*p* = 0.269			*p* = 1
No bone defect	19	19		12	16		7	3	
Bone defect	7	3		6	3		1	0	
Fracture instability			*p* = 0.43			*p* = 1			*p* = 0.28
Unilateral	15	13		14	8		4	2	
Bilateral	6	8		9	4		0	1	
Symphysis	5	1		1	1		4	0	
Weight bearing			*p* = 0.77			*p* = 1			*p* = 0.56
No weight-bearing	14	10		9	9		5	1	
Weight bearing	12	12		9	10		3	2	
Infra-acetabular screws			*p* = 0.44			*p* = 0.33			*p* = 1
No infra-acetabular screw	6	3		4	3		2	0	
Unilateral acetabular screw	10	6		9	6		1	0	
Supra-acetabular screws			*p* = 0.53			*p* = 0.30			*p* = 1
No supra-acetabular screw	17	17		11	15		6	2	
One or more supra-acetabular screws	9	5		7	4		2	1	
Fixation posterior pelvic ring			*p* = 0.7			*p* = 0.45			*p* = 1
No posterior fixation	5	3		5	3		0	0	
Posterior fixation	21	19		13	16		8	3	
FFP fracture type			*p* = 0.193			*p* = 0.092			*p* = 0.308
FFP1	2	0		2	0		0	0	
FFP2	5	6		2	6		3	0	
FFP3	7	2		6	2		1	0	
FFP4	12	14		8	11		4	3	
Type of posterior fixation			*p* = 0.32			*p* = 0.24			*p* = 0.9
Iliosacral screws	0	2		0	2		0	0	
Transsacral bar	6	5		3	4		3	1	
Transsacral bar + SI screw(s)	11	12		8	11		3	1	
Plate osteosynthesis ilium	3	2		3	2		0	0	
Transiliac internal fixator + iliosacral screws	4	1		2	0		2	1	

No SL (no screw loosening) and SL (screw loosening)

## Discussion

The goal of our study was to analyze the implant-related complications in SPO and DPO of the anterior pelvic ring in patients with FFP. We hypothesized that complications would be less frequent and less severe after DPO than after SPO. SL was seen in the superior plate in 45% of our patients and can therefore be regarded as very common. There was no SL in the second, anterior plate. SL was most often seen in the Nakatani I region. Not all SL led to the need for surgical revision. The limited need for surgical revision has also been described in patients, who were treated with SPO for traumatic disruption of the symphysis [10]. DPO led to a lower rate of SL, less severe SL and later onset of SL when compared with SPO. This could be explained by the reduced motion at the pubic symphysis when a DPO is used [16]. Plate breakage of SPO for high-energy traumatic rupture of the symphysis is not uncommon and described in up to 43% [15]. Plate breakage is rarer in our group of patients and we hypothesize that this could be due to the high rate of screw loosening, which prevents breakage. We performed revision osteosynthesis in six patients (6/48) and all of these were after SPO (6/37). Although hardware problems after fixation of the anterior pelvic ring and the symphysis pubis are common, revision surgery is rare [15]. In case of instability at the pubic symphysis, we significantly more often performed DPO with bilateral infra-acetabular screws at the margins of the pelvic brim plate [17, 18].

There are several alternatives to stabilize the anterior pelvic ring in FFP. The use of external fixation is controversial as it has the advantage that it can be easily applied and easily removed but provides less stability and causes severe discomfort [19, 20]. Retrograde transpubic screws can be easily applied in non-displaced pubic rami fractures and provide adequate stability to allow fracture healing in the fractures that are located in Nakatani zone II and zone III but are less suited for fractures in zone I [2]. The anterior subcutaneous crossover pelvic fixator or INFIX functions as an internal fixator with the connecting rod positioned subcutaneously. It combines the advantage of the easily placed supra-acetabular screws without the need of an external fixation frame. It can however cause some important complications, such as heterotopic ossifications, femoral nerve palsy and femoral artery occlusion  [21]. Plate osteosynthesis precludes fracture exposure but has the advantages of biomechanical superiority and minimal interference with the soft tissues [22]. Our results show that in osteoporotic bone, early loosening is a common problem but that his can be avoided using a double plate construct. Several authors performed biomechanical studies to assess which plate construct is optimal. Moed et al. could not find any difference in the occurrence of failure between locked and unlocked 6-hole symphysis plates in B and C type fractures in a double leg stance model [8, 23]. Godynski et al. noticed a small but increased stability when locking plate fixations are used in an increased load single limb stance model. Simonian et al. investigated the stability of box plate (double plate) fixation on symphysis ruptures and showed that this kind of configuration resulted in the least amount of motion at the level of the symphysis.

A common problem of symphysis crossing plate fixation is loosening of the screws [13]. Collinge et al. reported on fixation failure; including screw loosening in 75% of patients in a series of 127 adult patients with a mean age of 41 years and a follow-up of 6 months [10]. Eastmann et al. reported a fixation failure of 31% of which 11% occurred within the first 7 weeks after surgery. It can be expected that in patients with limited bone mineral density, problems, such as SL or implant failure, may be even more frequent. Our results show that the rate of SL and implant failure is within the same range as in patients with a high-energy disruption of the pubic symphysis [10, 13, 24]. We hypothesize that this is because of the intact ligamentous structures around the symphysis in FFP. The plates that were used in our study are longer than the ones used in the above mentioned biomechanical studies. The pubic bone is known to have less bone mineral density when compared with the posterior pelvis [25]. Using longer plates and longer screw trajectories, such as the infra-acetabular screws, which increase the plate fixation strength up to 50% in acetabular fractures, we aimed to increase the stability of our construct [18]. Longer plates, however, did not significantly influence the SL rate. DPO proved to achieve higher stability in the biomechanical study of Simonian et al. In our study, we found a lower rate of SL, less severe SL and later onset of SL in DPO. No revision surgery was needed after DPO. We therefore do support the use of DPO in FFP with anterior instability in or near to the pubic symphysis.

Posterior pelvic ring fixation did not significantly influence the rate of SL in our cohort. Avilucea et al. showed that posterior fixation in APC-2 injuries significantly reduces anterior plate failure [26]. These APC injuries however cannot be compared with FFP injuries as in FFP patients, there is no ligamentous disruption that causes increased instability. There is a lack of biomechanical studies that have evaluated the instability that is associated with FFP and further biomechanical research on this topic is necessary.

This study has several limitations. The main limitation is the limited number of patients and the size difference of the two cohorts. This also leads to a lack of statistical significance in our results. Nevertheless, as implant-related complications of plate fixation of the anterior pelvic ring in FFP have not been described yet, we do believe that there is some valuable data in our results. Another limitation is that the data was collected retrospectively with different follow-up periods of our patients. This is however a common problem in retrospective studies involving the geriatric population. The review was only focused on complications related to plate osteosynthesis, but not to general medical or surgical peri-operative complications. Outcome of treatment of FFP does not only depend on anterior plate fixation but is depending on many other factors.

Despite these limitations, the study offers valuable new information. It is, to our knowledge, the first study that analyses the implant-related complications related to SPO and DPO of the anterior pelvic ring in patients with FFP.

## Conclusion

Almost half of the patients with FFP, who were treated with a plate osteosynthesis of the anterior pelvic ring experienced SL at some point during follow-up. The vast majority of SL is situated near to the pubic symphysis. DPO leads to a lower rate of SL, less severe SL and later onset of SL when compared with SPO. Revision osteosynthesis was not needed after DPO. We therefore recommend DPO whenever plate osteosynthesis seems the best option for fixation of anterior pelvic ring in FFP. Further prospective and biomechanical studies are needed to gain more evidence on surgical stabilization of the anterior pelvic ring in FFP.

## Abbreviations

FFP: Fragility fracture of the pelvis
SPO: Single plate osteosynthesis
DPO: Double plate osteosynthesis
SL: Screw loosening
SD: Standard deviation

## References

[1] Rommens PM, Arand C, Hopf JC, Mehling I, Dietz SO, Wagner D (2019). Progress of instability in fragility fractures of the pelvis: an observational study. Injury.

[2] Rommens PM, Graafen M, Arand C, Mehling I, Hofmann A, Wagner D (2020). Minimal-invasive stabilization of anterior pelvic ring fractures with retrograde transpubic screws. Injury.

[3] Scheyerer MJ, Osterhoff G, Wehrle S, Wanner GA, Simmen H-P, Werner CML (2012). Detection of posterior pelvic injuries in fractures of the pubic rami. Injury.

[4] Rommens PM, Hofmann A (2013). Comprehensive classification of fragility fractures of the pelvic ring: recommendations for surgical treatment. Injury.

[5] Rommens PM, Arand C, Hofmann A, Wagner D (2019). When and how to operate fragility fractures of the pelvis?. Indian J Orthop.

[6] Van Loon P, Kuhn S, Hofmann A, Hessmann MH, Rommens PM (2011). Radiological analysis, operative management and functional outcome of open book pelvic lesions: a 13-year cohort study. Injury.

[7] Simonian P, Routt ML, Harrington R, Mayo K, Tencer A (1994). Biomechanical simulation of the anteroposterior compression injury of the pelvis: an understanding of instability and fixation. Clin Orthop Relat Res.

[8] Grimshaw CS, Gary Bledsoe J, Moed BR (2012). Locked versus standard unlocked plating of the pubic symphysis: A cadaver biomechanical study. J Orthop Trauma.

[9] Putnis SE, Pearce R, Wali UJ, Bircher MD, Rickman MS (2011). Open reduction and internal fixation of a traumatic diastasis of the pubic symphysis: One-year radiological and functional outcomes. J Bone Joint Surg Br.

[10] Collinge C, Archdeacon MT, Dulaney-Cripe E, Moed BR. Radiographic changes of implant failure after plating for pubic symphysis diastasis: An underappreciated reality? Trauma. Clin Orthop Relat Res. New York LLC: Springer; 2012. p. 2148–2153. 10.1007/s11999-012-2340-5 PMC3392370 22552765

[11] Archdeacon MT, Collinge CA, Schumaier AP, Glogovac G (2018). Effect of Deformity and Malunion of the Anterior Pelvic Ring. J Orthop Trauma.

[12] Collinge CA, Archdeacon MT, LeBus G (2009). Saddle-horn injury of the pelvis. The injury, its outcomes, and associated male sexual dysfunction. J Bone Joint Surg Am.

[13] Eastman JG, Krieg JC, Routt MLC (2016). Early failure of symphysis pubis plating. Injury.

[14] Starr AJ, Nakatani T, Reinert CM, Cederberg K (2008). Superior pubic ramus fractures fixed with percutaneous screws: what predicts fixation failure?. J Orthop Trauma.

[15] Simonian PT, Routt ML, Harrington RM, Tencer AF (1994). Box plate fixation of the symphysis pubis: biomechanical evaluation of a new technique. J Orthop Trauma..

[16] Morris SAC, Loveridge J, Smart DKA, Ward AJ, Chesser TJS. Is fixation failure after plate fixation of the symphysis pubis clinically important? Trauma. Clin Orthop Relat Res. New York LLC: Springer; 2012. p. 2154–2160. 10.1007/s11999-012-2427-z PMC3392398 22707071

[17] Culemann U, Marintschev I, Gras F, Pohlemann T (2011). Infra-acetabular corridor-technical tip for an additional screw placement to increase the fixation strength of acetabular fractures. J Trauma.

[18] Marintschev I, Gras F, Schwarz CE, Pohlemann T, Hofmann GO, Culemann U (2012). Biomechanical comparison of different acetabular plate systems and constructs - The role of an infra-acetabular screw placement and use of locking plates. Injury.

[19] Gänsslen A, Hildebrand F, Kretek C (2013). Supraacetabular external fixation for pain control in geriatric type B pelvic injuries - PubMed. Acta Chir Orthop Traumatol Cech.

[20] Mason WTM, Khan SN, James CL, Chesser TJS, Ward AJ (2005). Complications of temporary and definitive external fixation of pelvic ring injuries. Injury.

[21] Vaidya R, Martin AJ, Roth M, Tonnos F, Oliphant B, Carlson J. Midterm Radiographic and Functional Outcomes of the Anterior Subcutaneous Internal Pelvic Fixator (INFIX) for Pelvic Ring Injuries. J Orthop Trauma. Lippincott Williams and Wilkins; 2017. p. 252–259. 10.1097/BOT.0000000000000781 PMC5402711 28079731

[22] Acklin YP, Zderic I, Buschbaum J, Varga P, Inzana JA, Grechenig S (2016). Biomechanical comparison of plate and screw fixation in anterior pelvic ring fractures with low bone mineral density. Injury.

[23] Moed BR, O’Boynick CP, Bledsoe JG (2014). Locked versus standard unlocked plating of the symphysis pubis in a Type-C pelvic injury: a cadaver biomechanical study. Injury.

[24] Frietman B, Verbeek J, Biert J, Frölke JP (2016). The effect of implant failure after symphyseal plating on functional outcome and general health. J Orthop Trauma.

[25] Arand C, Wagner D, Richards RG, Noser H, Kamer L, Sawaguchi T (2019). 3D statistical model of the pelvic ring—a CT-based statistical evaluation of anatomical variation. J Anat.

[26] Avilucea FR, Whiting PS, Mir H (2016). Posterior fixation of APC-2 pelvic ring injuries decreases rates of anterior plate failure and malunion. J Bone JtSurg Am.

